# Hepatoprotective Effect and Synergism of Bisdemethoycurcumin against MCD Diet-Induced Nonalcoholic Fatty Liver Disease in Mice

**DOI:** 10.1371/journal.pone.0147745

**Published:** 2016-02-16

**Authors:** Sung-Bae Kim, Ok-Hwa Kang, Young-Seob Lee, Sin-Hee Han, Young-Sup Ahn, Seon-Woo Cha, Yun-Soo Seo, Ryong Kong, Dong-Yeul Kwon

**Affiliations:** 1 Department of Oriental Pharmacy, College of Pharmacy, Wonkwang University, Wonkwang Oriental Medicines Research Institute, Iksan, Jeonbuk, 570–749, Korea; 2 BK21 Plus Team, Professional Graduate School of Oriental Medicine, Wonkwang University, Iksan, Jeonbuk, 570–749, Korea; 3 Department of Herbal Crop Research, National Institute of Horticultural & Herbal Science, RDA, 92 Bisanro, Eumsung, Chungbuk, 369–873, Korea; University of Catania, ITALY

## Abstract

Nonalcoholic fatty liver disease (NAFLD), the hepatic manifestation of the metabolic syndrome, has become one of the most common causes of chronic liver disease over the last decade in developed countries. NAFLD includes a spectrum of pathological hepatic changes, such as steatosis, steatohepatitis, advanced fibrosis, and cirrhosis. Bisdemethoxycurcumin (BDMC) is polyphenolic compounds with a diarylheptanoid skeleton, curcumin close analogues, which is derived from the *Curcumae Longae* Rhizoma. While the rich bioavailability research of curcumin, BDMC is the poor studies. We investigated whether BDMC has the hepatoprotective effect and combinatory preventive effect with silymarin on methionine choline deficient (MCD)-diet-induced NAFLD in C57BL/6J mice. C57BL/6J mice were divided into five groups of normal (normal diet without any treatment), MCD diet (MCD diet only), MCD + silymarin (SIL) 100 mg/kg group, MCD + BDMC 100 mg/kg group, MCD + SIL 50 mg/kg + BDMC 50 mg/kg group. Body weight, liver weight, liver function tests, histological changes were assessed and quantitative real-time polymerase chain reaction and Western blot analyses were conducted after 4 weeks. Mice lost body weight on the MCD-diet, but BDMC did not lose less than the MCD-diet group. Liver weights decreased from BDMC, but they increased significantly in the MCD-diet groups. All liver function test values decreased from the MCD-diet, whereas those from the BDMC increased significantly. The MCD- diet induced severe hepatic fatty accumulation, but the fatty change was reduced in the BDMC. The BDMC showed an inhibitory effect on liver lipogenesis by reducing associated gene expression caused by the MCD-diet. In all experiments, the combinations of BDMC with SIL had a synergistic effect against MCD-diet models. In conclusion, our findings indicate that BDMC has a potential suppressive effect on NAFLD. Therefore, our data suggest that BDMC may act as a novel and potent therapeutic agent against NAFLD.

## Introduction

Nonalcoholic fatty liver disease (NAFLD) affects a large population in the world [1]. It is primarily associated with the metabolic syndrome including insulin resistance, diabetes, obesity, hyperlipidemia [2] [3]. Especially, obesity is an alarming public health big issue because it causes metabolic syndromes, such as type 2 diabetes, hypertension, cardiovascular disease, NAFLD, and insulin resistance [4]. Hepatic steatosis is defined by the presence of cytoplasmic triglyceride (TG), droplets in > 5% of hepatocytes in the absence of significant alcohol consumption [5]. It is recognized as a decisive “first-hit” in the pathogenesis of liver disease [5]. But, the “first hit” sensitives the liver to injury mediated by the “second hit”, such as adipokines, oxidative stress, inflammatory cytokines and mitochondrial dysfunction, leading to nonalcoholic steatohepatitis (NASH) [6]. NAFLD, there is a tendency to develop in obese or diabetic patients. It has most of the adult liver steatosis of obesity, at least, one third of these individuals will develop a worsening NAFLD [7] [8]. In addition, the prevalence of NAFLD will likely to increase obesity rate.

Recently, natural herbs and food material have been the focus of many researchers because of their safety and efficacy, and potential bio-active ingredient to prevent or treat obesity and NAFLD [9] [10]. *Curcumae longae* rhizoma is a widely used traditional herb in many countries, and contains spice and yellow flavoring agent from the root of *Curcuma longa* L. [11]. It has been traditionally used to various diseases, such as hyperlipidemia, cancer, stomach ache, diabetes mellitus, wounds, and hepatic disorders [12]. The main constitute of curcuma is curcumin, which constitutes up to 90% of total curcuminoid content, with demethoxycurcumin and bisdemethoxycurcumin (BDMC) comprising the remainder [11]. This plant polyphenolic compound has anti-tumor, anti-proliferative, anti-oxidant, anti-fungal, anti-hepatotoxic, anti-diabetic and anti-inflammatory activities, as well as some side effects [12]. In particular, curcumin has been demonstrated anti-adipogenic effect in 3T3-L1 cell model [13] [14]. Pharmacological studies have compared the efficacy with steatohepatitis induced by a methionine and choline deficient (MCD)-diet model and HepG2 cells model [15]. However, the mechanism by which BDMC exerts its hepatoprotective effects has not yet been fully uncovered. Silymarin is a polyphenolic flavonoid isolated from milk thistle *Silybum marianum*, it’s widely used liver protective agent against hepatotoxicity. So, we evaluated the efficacy of BDMC in preventing steatohepatitis in mice and investigated the underlying mechanism. Also, we tested whether BDMC has combinatory hepatoprotective effect with silymarin on MCD-diet mice model.

## Materials and Methods

### Materials

**Fig 1:**
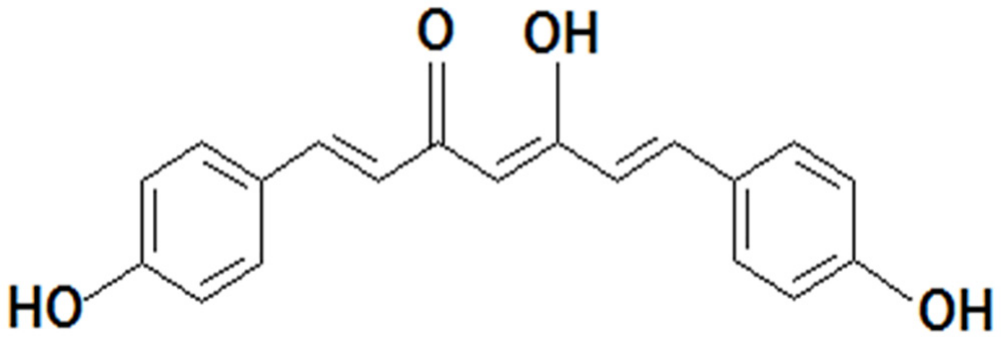
Chemical structure of BDMC.

Bisdemethoxycurcumin (BDMC, Fig 1) was purchased from TCI America (Portland, OR, USA). Silymarin (SIL) was purchased from Sigma-Aldrich (St Louis, MO, USA). Anti-β-actin, peroxisome proliferator activated receptor (PPAR)-α, γ, CCAAT/enhancer binding protein (C/EBP) α, sterol regulatory element binding protein (SREBP), fatty acid synthase (FAS) antibodies were obtained from Santa Cruz Biotechnology (Santa Cruz, CA, USA). Interleukin (IL)-6 and tumor necrosis factor (TNF)-α antibodies and biotinylated antibodies for mouse IL-6 and TNF-α were purchased from BD Biosciences (San Jose, CA, USA). Anti-pThr172- 5' AMP-activated protein kinase (AMPK) and anti-AMPK antibodies were purchased from Cell Signaling Technology (Beverly, MA, USA). SREBP-1c, PPAR-α, PPAR-γ, FAS, and GAPDH oligonucleotide primers were purchased from Bioneer Corp. (Daejeon, Korea).

### Animal care and diet preparation

Seven-week-old male C57BL/6J mice were obtained from Samtaco Korea (Seoul, Korea). The mice were given free access to water and kept at a constant room temperature under a 12/12-hour light/dark cycle. They were allowed to adapt to their food and environment for 1 week before starting the experiment. The C57BL/6 mice were divided into 5 groups (7 mice per group) and its group shown in Table 1. Namely, normal diet (Dyets Inc., Bethlehem, PA, USA), MCD diet (Dyets Inc., Bethlehem, PA, USA), SIL 100 (MCD diet supplemented with silymarin 100 mg/kg/day), BDMC 100 (MCD diet supplemented with BDMC 100 mg/kg/day), SIL 50+BC 50 (MCD diet supplemented with silymarin 50 mg/kg/day + BDMC 50 mg/kg/day) was orally administered by gavages to the mice daily during the 4 weeks of diet feeding. The composition of the experimental diet was shown in Table 2. After four weeks, animals were sacrificed *via* CO_2_ inhalation for the collection of blood and liver samples. The investigation conforms to the Guide for the Care and Use of Laboratory Animals published by the US National Institute of Health (NIH Publication No. 85–23, revised 1996) and was approved by the Institutional Animal Care and Utilization Committee for Medical Science of Wonkwang University (Approval no.WKU-15-100).

**Table 1:** The experimental groups.

Mouse model	Treatment (mg/kg/day)	
C57BL/6J	Normal	Normal
	MCD	MCD
	MCD+Silymarin (100 mg)	SIL 100
	MCD+BDMC (100 mg)	BDMC 100
	MCD+SIL (50 mg)+BDMC (50 mg)	SIL 50+BC 50

**Table 2:** Constituents of experimental diet.

Ingredient	Normal diet (gm/kg)	MCD-diet (gm/kg)
L-Arginine	16.2	12.7
L-Histidine	6.4	3.4
L-Lysine HCl	13.8	9.1
L-Tyrosine	8.7	5.7
L-Tryptophan	2.8	1.8
L-Phenylalanine	11.8	7.3
L-Methionine	4.4	0
L-Cystine	3.9	3.7
L-Threonine	9.2	4.6
L-Leucine	20.2	10.5
L-Isoleucine	8.8	6.1
L-valine	11.7	6.3
Glycine	0	6.2
L-Proline	0	7.6
L-Glutamic Acid	0	28.9
L-Alanine	0	5.1
L-Aspartic Acid	0	15.8
L-Serine	0	7.2
Cornstarch	100	100
Dextrin	100	100
Sucrose	408.58	408.58
Celluose (401855)	50	50
Corn Oil	50	50
SaltMix20000	35	3.5
Sodium Bicarbonat	4.3	4.3
choline bitartrate	2	0
VitaminMix 300050	10	10
Primex	100	100
FerricCitrate, U.S.P.	0.12	0.12
Total	1000.0	1000.0

### Histological examination

The formalin was exchanged for fresh solution of the liver slices fixed in 10% formalin (paraformaldehyde [Junsei Chemical Co., Ltd, Tokyo, Japan] and phosphate-buffered saline [PBS, pH 7.4]) overnight. Each formalin-fixed liver sample was embedded in paraffin and sliced into 4-μm-thick sections. The slides were stained with hematoxylin and eosin (H&E) and evaluated by three investigators.

### Biochemical analysis

Serum TG, total cholesterol (TC), alanine aminotransferase (ALT) and aspartate aminotransferase (AST) were estimated using a commercial enzymatic kit (Asan, Seoul, Korea). As described in detail, liver was homogenized in 0.5 mL 1 M NaCl. The liver tissue homogenate was extracted with 3 mL chloroform/methanol (2:1) plus 0.5 mL 1 M NaCl. The organic phase was collected, dried, and resuspended in 0.5 M Triton X-100/methanol (2:1). Hepatic TG, TC, ALT, and AST were determined using a commercial enzymatic kit (Asan, Seoul, Korea).

### Cytokine release analysis

Blood samples were collected after an 18 h overnight fast in sacrificed animals to determine the IL-6 and TNF-α concentration. Peripheral serum was subjected to enzyme-linked immunosorbent assay (ELISA) using IL-6 and TNF-α kit BD Biosciences (San Jose, CA, USA). Absorbance was read at 450 nm using a microplate reader (Biotec, Chicago, IL, USA). Samples and standards were run three times.

### Western blot analysis

Protein expression was assessed by Western blot analysis according to standard procedures. Namely, the liver was homogenized in RIPA lysis buffer (iNtRON biotech, Daejon, Korea) on ice. The homogenates were centrifuged (13,000 rpm, 10 min, 4°C), and the protein concentrations in the supernatant were determined using the Bio-Rad protein assay reagent (Bio-Rad Laboratories, Hercules, CA, USA) according to the manufacturer’s instructions. Equal amounts of protein (20 μg) were subjected to sodium dodecyl sulfate-polyacrylamide gel electrophoresis and transferred to a polyvinylidene membrane (Millipore, Bedford, MA, USA). The membrane was blocked for 1 hour with 5% skim milk in Tris-buffered saline buffer (150 mM NaCl and 20 mM Tris-HCl, pH 7.4) with 0.05% Tween 20. The membrane was incubated with primary antibodies for 18 h, washed with Tris-buffered saline with Tween 20, and incubated with anti-mouse or anti-rabbit immunoglobulin G horseradish peroxidase-conjugated secondary antibodies. The proteins were supplemented with the ECL prime Western blotting detection reagents (GE Healthcare, Parsippany, NJ, USA) and ImageQuant LAS 4000 Mini Biomolecular Imager (GE Healthcare, Parsippany, NJ, USA) was used to evaluate the bands, which were quantified by Image j.

### Quantitative real-time polymerase chain reaction analysis (qRT-PCR)

Total-RNA was extracted from livers using an easy-BLUE total-RNA extraction kit according to the manufacturer’s instructions. Single-strand cDNA synthesis was performed using the Quantitact reverse transcription kit according to the manufacturer’s instructions. The RT-PCR analysis was performed with a QuantiTect^™^ SYBR Green PCR. The RT-PCR data were based on SYBR green amplification. The primer sequences are listed in Table 3. mRNA was detected for PPARα, PPARγ, SREBP-1c, Fas, C/EBPα, and GAPDH using the LightCycler system (Bio-Rad, Hercules, California, U.S.A.). Each sample was run and analyzed in duplicate.

**Table 3:** Primer sequences for real-time RT-PCR.

Target genes	Primer sequences	
	Forward primer	Reverse primer
SREBP-1c	CATCGACTACATCCGCTTCTTACA	GTCTTTCAGTGATTTGCTTTTGTGA
PPARα	TGGAGTCCACGCATGTGAAG	CGCCAGCTTTAGCCGAATAG
C/EBP	GCC GAG ATA AAG CCA AAC AA	CCT TGA CCA AGG AGC TCT CA
PPARγ	TTT TCA AGG GTG CCA GTT TC	TTA TTC ATC AGG GAG GCC AG
FAS	TGGTGGGTTTGGTGAATTGTC	GCTTGTCCTGCTCTAACTGGAAGT
GAPDH	AAC TTT GGC ATT GTG GAA GG	GGA TGC AGG GAT GAT GTT CT

### Statistical analysis

The statistical analysis was performed with one-way analysis of variance using IBM SPSS Statistics 19 (IBM Corp., Armonk, NY, USA). Data are presented as means ± standard deviations.

## Results

### Effects of BDMC on body weight and liver index of mice fed with MCD diet

Body weight was measured from the beginning and the end of the experiment, at the terms of 2 days. Mice fed the MCD diet lost significant body weight compared with mice fed the control diet. The same observations were made for liver weight (Table 4).

**Table 4:** Effect of BDMC on MCD diet body weight and liver weight.

	Normal (mg/kg/day)	MCD (mg/kg/day)	MCD+ SIL 100 (mg/kg/day)	MCD+ BDMC 100 (mg/kg/day)	MCD+ SIL 50+BC 50 (mg/kg/day)
Body weight (g)					
Initial	22.2±2.2	22.1±2.6	23.6±3.6	23.8±1.5	22.9±3.5
Final	25.5±2.3	15.2±1.7^a^	17.3±1.6*	19.4±1.5**	18.6±4.3**
Liver weight (g)	0.89±0.02	1.36±0.06^a^	0.85±0.03**	0.51±0.06**	0.65±0.02**

Data are expressed as mean ± SD from 7 animals where ap < 0.05 as compared to normal;

*p < 0.05,

**p < 0.01, as compared with the MCD group.

### Effects of BDMC on circulating ALT and AST levels

**Fig 2:**
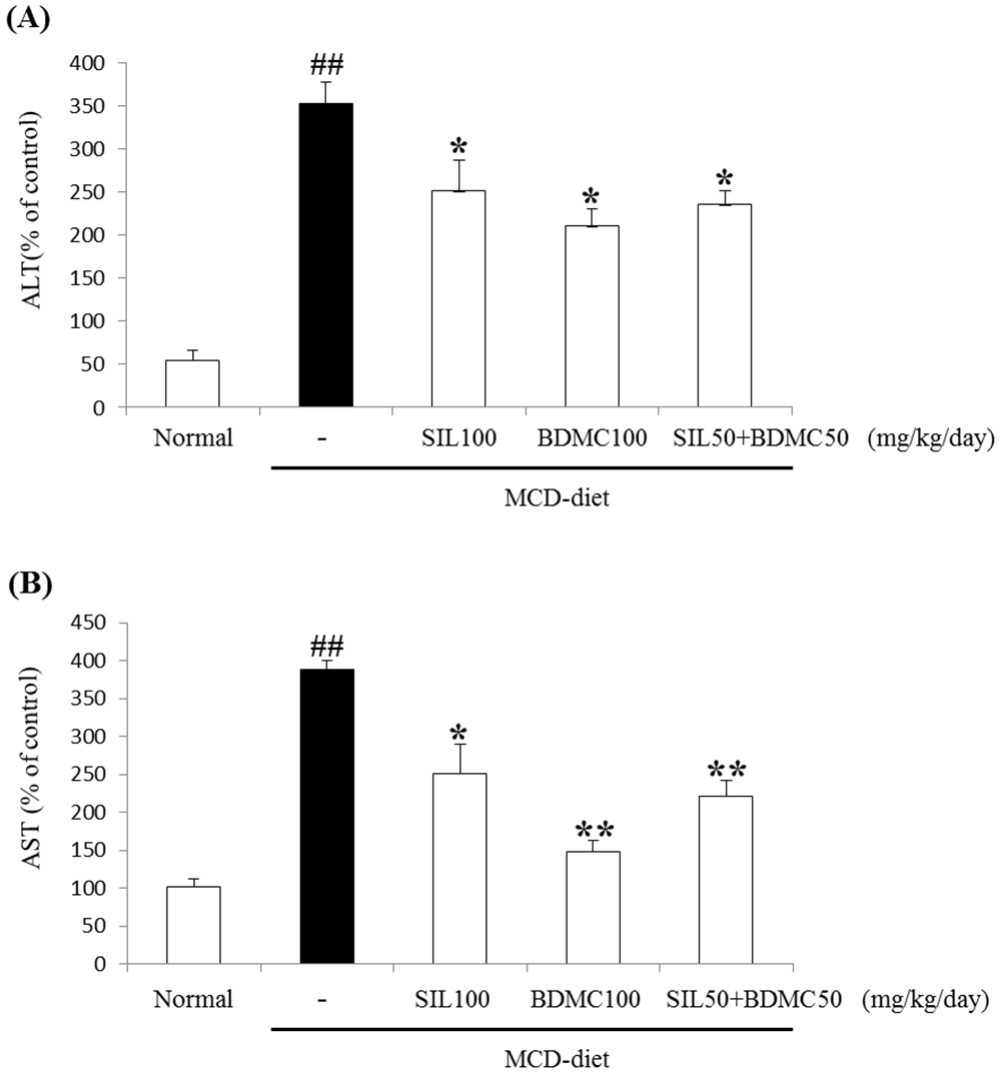
Effects on ALT and AST levels in mice. Mice were fed a control diet or the MCD diet for 4 weeks. Blood samples were collected, and plasma ALT (A) and AST (B) levels were determined. Mean ± standard deviation from seven animals is presented. ##p < 0.01 vs. normal control; *p < 0.05, **p < 0.01, vs. the MCD group.

Mice fed the MCD diet for 4 weeks developed severe steatohepatitis, with an associated elevation in the plasma AST and ALT. Circulating ALT and AST levels are a consequence of hepatocyte damage in NAFLD. Circulating ALT level decreased in the BDMC 100 and SIL 50 + BC 50 treated groups compared to that in the MCD-diet mice. Respectively, treatment with BDMC 100 inhibited this elevation in the plasma ALT and AST concentration (Fig 2).

### Effects of BDMC on histological evaluation of hepatic steatosis

**Fig 3:**
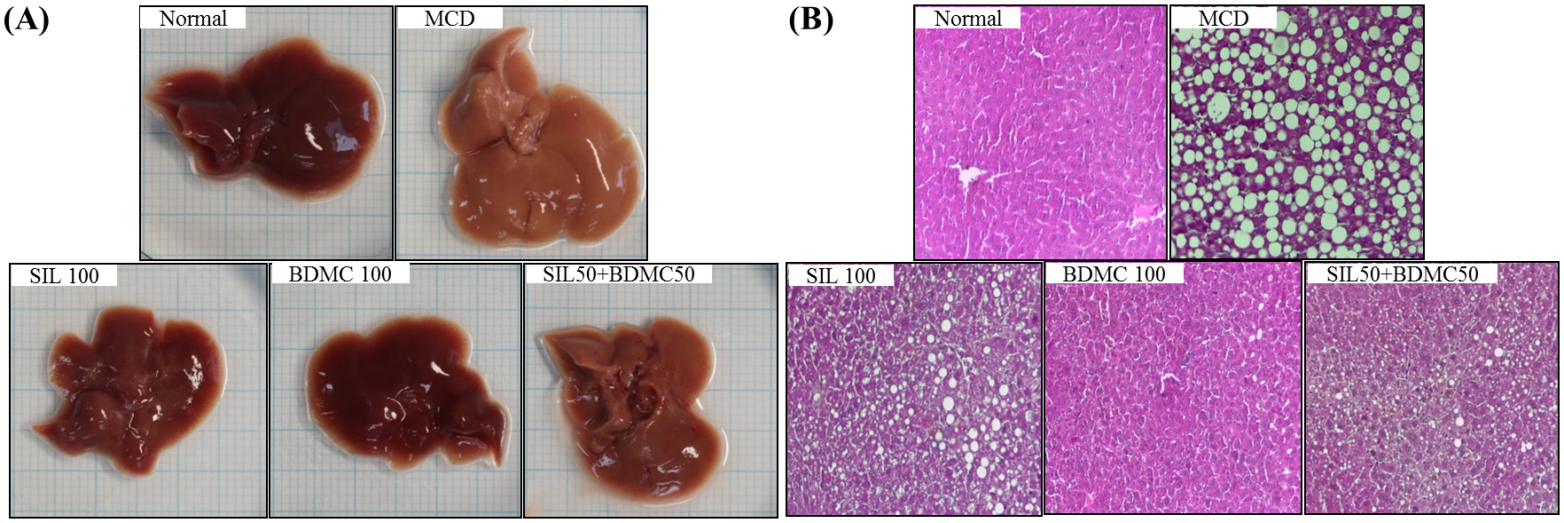
Histological analysis of liver steatosis and liver morphology. (A) Photographs of mice liver are shown. (B) C57BL/6J mice were fed a normal diet, the MCDdiet, or the same MCD diet supplemented with either treatment for 4 weeks. Liver sections were stained with hematoxylin and eosin. Original magnification, ×100 (B).

Hepatic steatosis appears excess lipid accumulation in hepatic parenchymal cells. Hepatic steatosis manifests as an accumulation of large macrovesicular or small microvesicular intracytoplasmic lipid droplets in hepatocytes. The diagnosis of steatosis is made when lipid content in the liver exceeds 5% by weight. The hallmark feature of NAFLD is steatosis. We examined the intrahepatic TG content in C57BL/6J mice to determine whether BDMC 100 and SIL 50 + BDMC 50 affected MCD-induced hepatic steatosis. Intrahepatic TG content increased in MCD-diet mice compared with that in normal mice (Fig 3). However, the BDMC 100 and SIL 50 + BDMC 50 treated mice showed lower intrahepatic TG contents than that of the MCD-diet mice. In particular, steatosis in the BDMC 100-treated mice completely almost disappeared.

### Effects of BDMC on TG and TC accumulation induced by MCD

We determined serum and liver TG and TC levels to examine the effect of BDMC on biochemical changes. The TG and TC levels increased significantly in MCD group compared to those in the control group. The BDMC 100 and SIL 50 + BDMC 50 groups showed significantly lower serum and liver TG and TC levels (Table 5).

**Table 5:** Biochemical liver function effects of the MCD-diet in C57BL/6J mice.

	Normal (mg/kg/day)	MCD (mg/kg/day)	MCD BDMC100 (mg/kg/day)	MCD SIL50+BC50 (mg/kg/day)
Serum (mg/L)				
TC	53.1±2.5	87.5±2.5 ^a^	40.5±1.2**	45.5±2.5*
TG	55.2±1.5	93.2±8.7 ^a^	48.2±1.5**	53.6±5.2**
HDL	45.5±1.4	27.5±6.5 ^a^	43.2±3.2*	42.5±2.6*
LDL	18.6±2.5	78.6±2.5^a^	6.9±0.8**	13.7±1.2**
Liver (mg/total tissue)				
TC	35.5±5.2	39.2±6.13	38.4±3.1	39.5±1.6
TG	39.7±4.3	108.8±5.1^a^	42.5±7.5**	62.8±6.4*
HDL	28.6±4.5	26.6±1.25	27.2±4.2	26.3±1.2
LDL	14.8±5.2	34.3±1.2^a^	19.7±2.3*	25.7±5.2

The actual values of mean ± SD from 7 animals are presented. ap < 0.05 as compared to normal;

*p < 0.05,

**p < 0.01, as compared with the MCD group.

**MCD;** methionine-choline.deficient diet, **TG;** triglyceride, **TC;** total cholesterol, **HDL;** high-density lipoprotein, **LDL;** low-density lipoprotein.

### Effects of BDMC on hepatic lipid accumulation and protein expression

**Fig 4:**
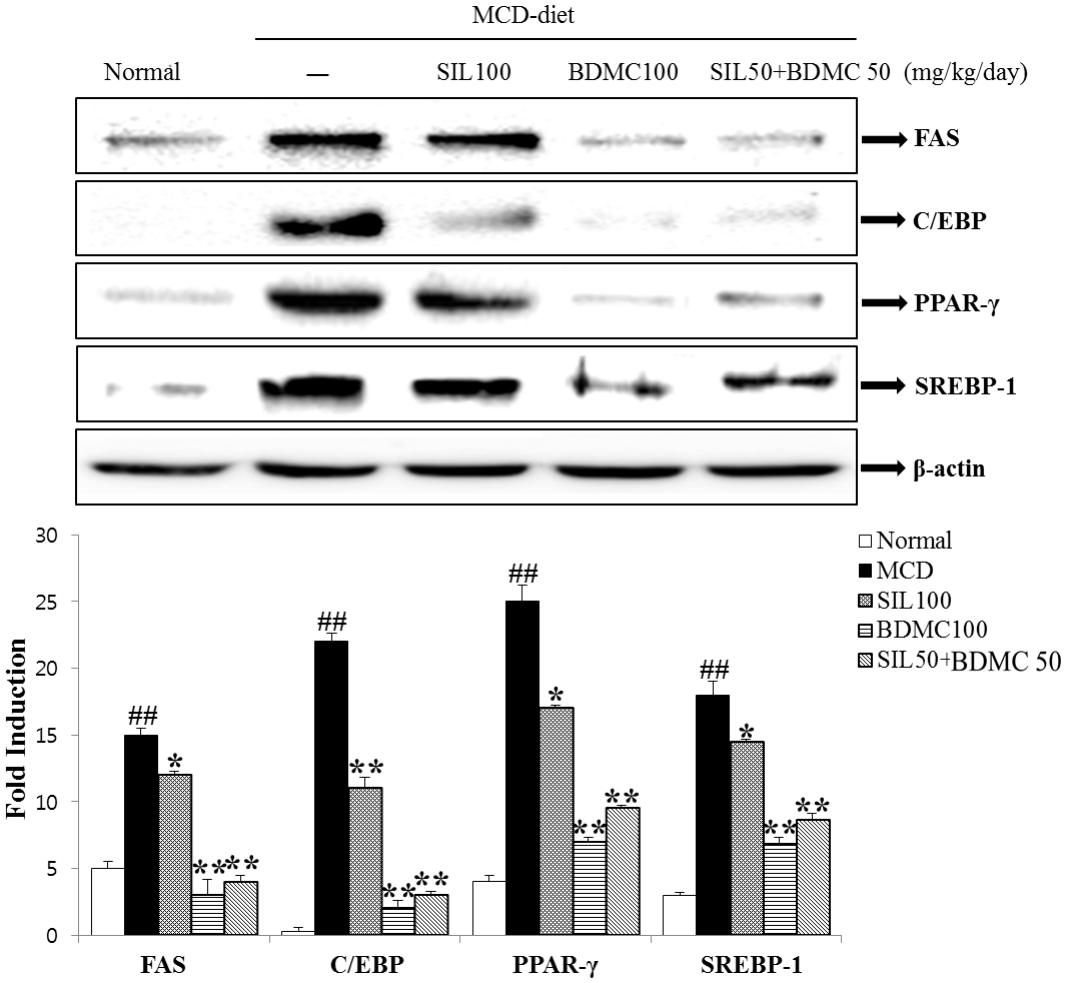
Effects on hepatic lipid accumulation and protein expression. PPAR-γ, C/EBPα, SREBP-1c, and FAS protein expression levels were detected by Western blot analysis. Expression levels were normalized to those of the β-actin protein. Mean ± standard deviation from seven animals is presented. ##p < 0.01 vs. normal; *p < 0.05, **p < 0.01 vs. control.

A Western blot analysis was performed the expression of adipogenic transcription factors and enzymes. The increases in FAS, C/EBPα, PPARγ, and SREBP-1 were suppressed significantly after treatment with BDMC 100 and SIL 50 + BDMC 50 (Fig 4). The FAS, C/EBPα, PPARγ, and SREBP-1 expression levels decreased.

### Effects of BDMC on hepatic lipogenic gene mRNA expression

**Fig 5:**
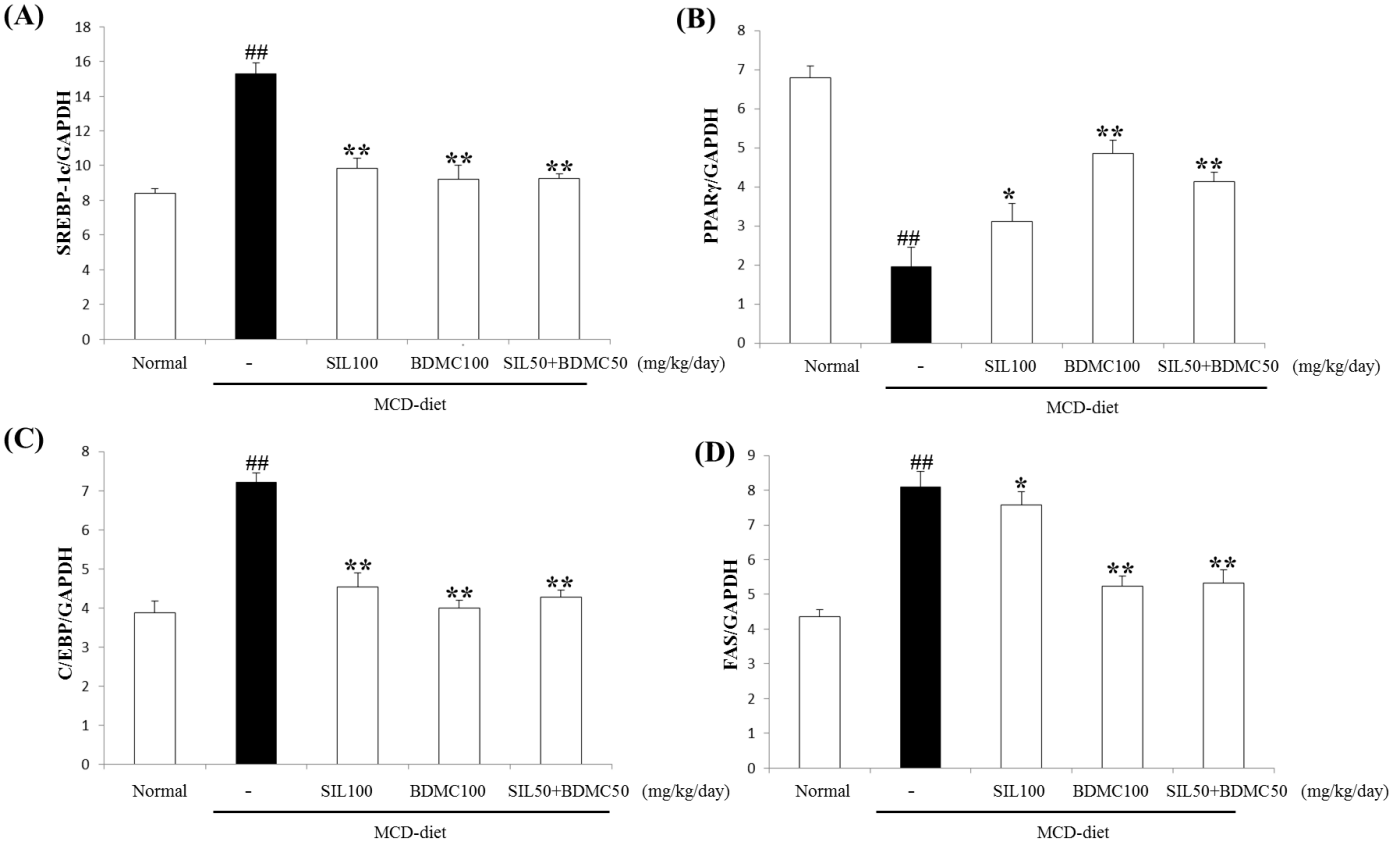
Hepatic lipogenesis gene mRNA expression levels. Data are representative of three independent experiments and quantified by densitometric analysis. mRNA expression levels were evaluated by real-time polymerase chain reaction and normalized to GAPDH levels. Data are mean ± standard deviation (n = 7, each). ##p < 0.01 vs. normal; *p < 0.05, **p < 0.01 vs. control.

Excess accumulation of stored lipid often leads to disorders, such as obesity and NAFLD. Gene related to fatty acid synthesis are generally upregulated in NAFLD. Hepatic lipogenesis rates are controlled by key transcription factors and metabolic enzymes, including SREBP1c and FAS. We measured the lipogenic gene mRNA levels to determine whether BDMC 100 and SIL 50 + BC 50 inhibited their expression. SREBP1c, PPARγ, C/EBP, and FAS mRNA levels in the BDMC 100 and SIL 50 + BDMC 50-treated mice decreased compared to those in MCD mice (Fig 5).

### Effects of BDMC on hepatic fatty acid oxidation and expression

**Fig 6:**
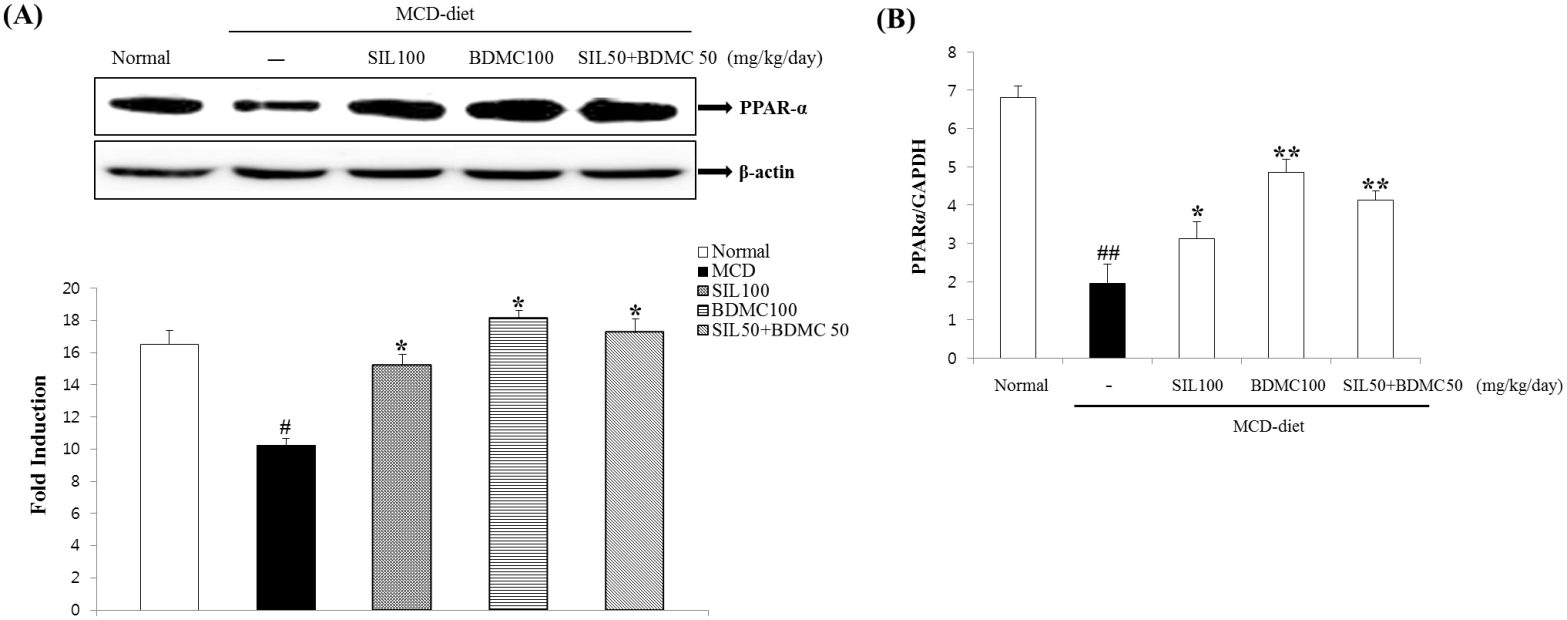
Effects on hepatic fatty acid oxidation and expression. (A) PPARα protein expression was detected by Western blot analysis. Expression levels were normalized to those of the β-actin protein. Mean ± standard deviation from seven animals is presented. #p < 0.05 vs. normal; *p < 0.05 vs. control. (B) Fatty acid oxidation gene mRNA expression. Data were representative of three independent experiments and quantified by densitometric analysis. mRNA expression levels were evaluated by real-time polymerase chain reaction and normalized to GAPDH levels. Data are mean ± standard deviation (n = 7 each). ##p < 0.01 vs. normal; *p < 0.05, **p < 0.01 vs. control.

Western blot analysis was performed to measure the expression of β-oxidation transcription factors and enzymes to demonstrate the effects of BDMC 100 and SIL 50 + BDMC 50 on fatty acid oxidation and protein levels. PPARα expression increased significantly after the BDMC 100 and SIL 50 + BDMC 50 treatments (Fig 6A). Also, we measured fatty acid oxidation gene mRNA expression to investigate the molecular hepatic lipid metabolic mechanism after the BDMC 100 and SIL 50+BDMC 50 treatments. Hepatic PPARα and its target enzymes are responsible for hepatic fatty acid oxidation. PPARα is a mitochondrial regulatory enzyme that transfers fatty acids from the cytosol to the mitochondria prior to β-oxidation. PPARα expression increased significantly after the BDMC 100 and SIL 50 + BDMC 50 treatments (Fig 6B).

### Effects of BDMC on MCD diet-induced hepatic inflammation

**Fig 7:**
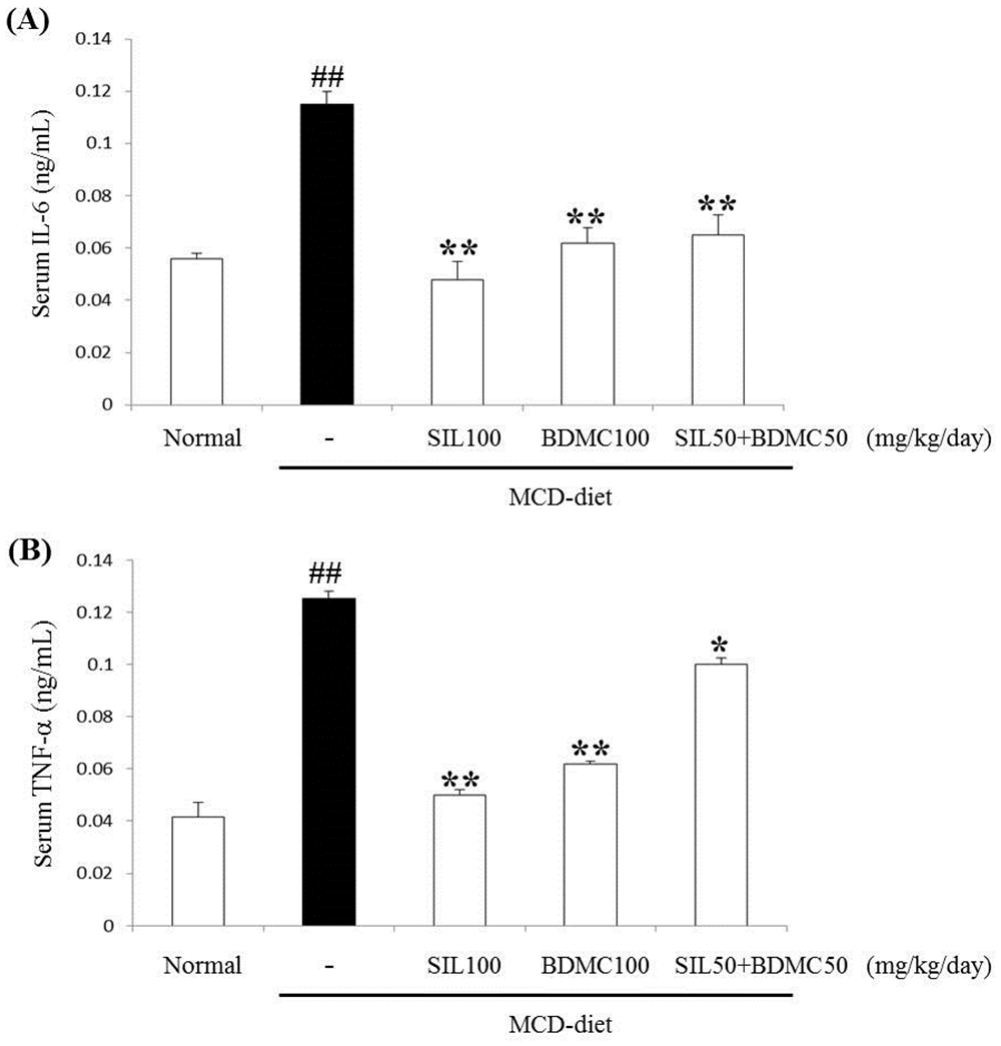
Effects on AMPK phosphorylation. AMPK phosphorylation (pThr-172-AMPK) was detected by Western blot analysis. Expression levels were normalized to that of the AMPK protein. Numbers below the panels represent quantification of the Western blot by densitometry. Mean ± standard deviation are presented (n = 7). ##p < 0.01 vs. normal; *p < 0.05, **p < 0.01 vs. control.

The inhibitory activities of the BDMC 100 and SIL 50 + BDMC 50 on TNF-α and IL-6 levels were tested with an ELISA. As shown in Fig 7, in serum TNF-α and IL-6 concentrations increased significantly on the MCD group. In addition, TNF-α and IL-6 concentrations decreased in mice treated with BDMC 100 and SIL 50 + BDMC 50, compared with that in the MCD group, suggesting that the BDMC 100 and SIL 50 + BDMC 50 treatments markedly inhibited TNF-α and IL-6 secretion.

### Effects of BDMC on AMPK phosphorylation

**Fig 8:**
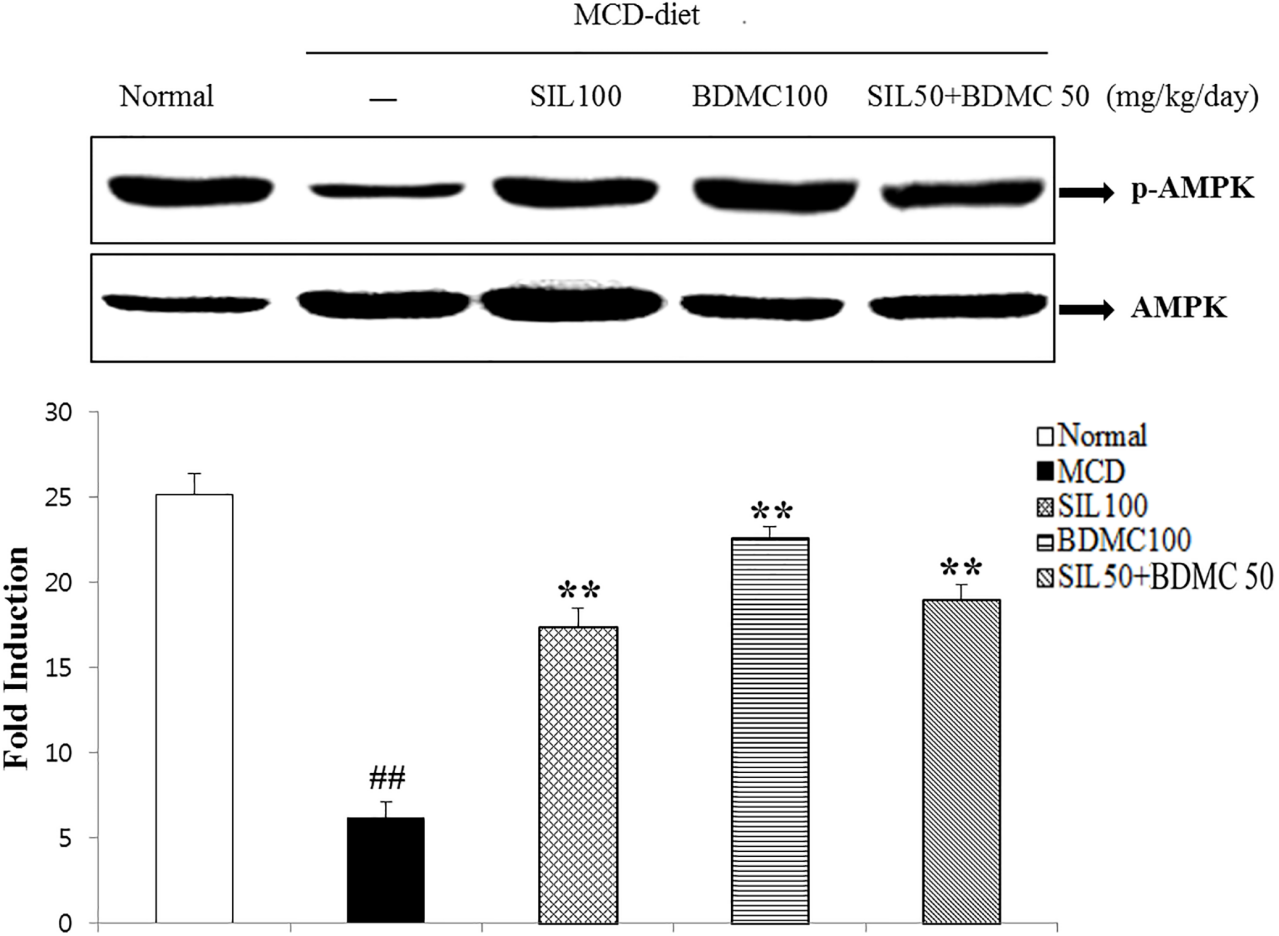
Effects on IL-6 and TNF-α concentrations. Mice were fed a control diet or the MCD diet for 4 weeks. Blood samples were collected, and plasma IL-6 (A) and TNF-α (B) concentrations were determined. Mean ± standard deviation are presented (n = 7). ##p < 0.01 vs. normal; *p < 0.05, **p < 0.01 vs. control.

We examined the effect of the BDMC 100 and SIL 50 + BDMC 50 treatments on AMPK phosphorylation of liver proteins to examine whether they activate the AMPK pathway. Phosphorylated AMPK decreased in the MCD group but increased significantly in the BDMC 100, and SIL 50+ BDMC 50 groups compared to that of the MCD group (Fig 8). Thus, the BDMC 100 and SIL 50 + BDMC 50 treatments may have a beneficial effect on NAFLD induced by MCD.

## Discussion

NAFLD is a liver symptom of metabolic syndrome. It has become one of the most common causes of chronic liver disease over the past 10 years in developed countries [16]. NAFLD contains a spectrum of pathological hepatic changes, such as steatohepatitis, steatosis, advanced fibrosis, and cirrhosis [17]. Feeding mice a methionine and choline deficient (MCD) diet leads to the development of steatohepatitis with fibrosis and serves an animal model for NAFLD [18]. The MCD diet is essential for hepatic β-oxidation and production of very low density lipoproteins (VLDL), choline deficiency impairs hepatic VLDL secretion [18]. Consequentially, lipid is accumulated in the liver. In addition, cytokines changes, oxidative stress, adipocytokines occur, contributing to the liver injury [19] [20].

*Curcumae longae* rhizoma is a plant in the Zingiberaceae family that provides a yellow flavorful powder when dried and ground. It is valued worldwide as a functional food because of its health promoting properties [21]. Several reports have indicated a variety of pharmacological activities of turmeric, such as antimicrobial, antiparasitic, antimutagenic, anticancer, antioxidant, anti-inflammatory and anti-human immunodeficiency virus [22–25]. It is effective for treating liver diseases, circulatory problems, and dermatological disorders [26–28]. The pharmacological activities of turmeric have been attributed to the ethanol extract, which contain three different curcuminoid pigments form the yellow color of turmeric, consist of curcumin, methoxy curcumin, demethoxy curcumin, and BDMC [29]. A previous study reported a hepatoprotective action of *Curcumae longae* rhizoma [14]. Additional studies have demonstrated that curcumin, demethoxycurcumin and BDMC have hepatoprotective activities [15]. However, no information is available about the effect of BDMC on NAFLD. In this study, we examined the hepatoprotective effect and underlying mechanism of BDMC in MCD-diet mice. Additionally, we tested BDMC may be used liver potent agents to be used in combating drug on NAFLD. Also, Silymarin (SIL) has been demonstrated that improves hepatic and myocardial injury in experimental nonalcoholic fatty liver disease and it used as a positive control [30]. To investigate the synergistic activity of BDMC with SIL, we used a half of capacity.

The MCD- diet model is associated with loss of body weight [31]. Thus, mice fed with the MCD diet lost significant body weight compared with mice fed the normal diet. However, adding BDMC to the MCD diet did not lead to further weight loss (Table 4). Silymarin (SIL) used positive control. The MCD diet also increased hepatic TG and serum ALT levels. But supplementing with MCD+BDMC group markedly alleviated hepatic TG accumulation (Table 5), high serum ALT and AST level (Fig 2), histological findings (Fig 3). In particular, the BDMC 100 group had significantly reduced values for these factors and SIL 50+ BDMC 50 group had a synergistic effect against MCD-diet models. Therefore BDMC protected the liver from damage when administered alone or in combination with SIL.

Proinflammatory cytokines mediate the inflammatory response and apoptosis [32] [33]. Especially, TNF-α has plays an important role in evolution of the steatohepatitis [34]. IL-6 is an inflammatory mediator of liver diseases, including obesity-associated fatty liver and cirrhosis [35] [36]. In our study, BDMC alone or in combination with SIL significantly suppressed oxidative stress and reduced TNF-α and IL-6 expression. It’s suggesting that the anti-inflammatory effects of BDMC may be partly related to inhibiting hepatic lipoperoxides and the expression of TNF-α and IL-6 (Fig 7).

Also, AMPK controls the white adipose tissue metabolism, acts as a “metabolic regulator”. AMPK suppresses energy consumption, such as sterol synthesis and fatty acid in the biosynthetic pathways, and activated ATP-producing catabolic pathways. AMPK has been implicated that hepatic glucose and lipid homeostasis control through genes and by short-term regulation of specific enzymes [37]. Our results suggest that BDMC alone or in combination with SIL may have a suppressive effect on MCD-diet induced lipid accumulation in the liver by activating AMPK phosphorylation (Fig 8). FAS is an enzyme necessary for *de novo* fatty acid synthesis, which is regulated by SREBP-1c [38] [39]. Reduction in fatty acid synthesis was considered a protective response against hepatosteatosis. In Figs 4 and 5, BDMC alone or in combination with SIL treatment reduced SREBP-1c and FAS protein and mRNA levels. Feeding the MCD diet resulted in a significant increase of PPARγ expression, which may promote elimination of fatty acids, reduce free fatty acid uptake by the liver, and lower inflammation [40]. PPARα is essential for the metabolism regulation and lipid transport, mainly by peroxisomal fatty acid β-oxidation and mitochondria activation pathways [41]. Especially, PPARα protects against high-fat-diet or MCD-diet induced NASH in rodents [42–44]. The BDMC alone or in combination with SIL showed reduced PPARγ and enhanced hepatic PPARα expression (Figs 4, 5 and 6). We propose that BDMC through AMPK shutting down the anabolic pathway and promoting catabolism by upregulating PPARα and downregulating the activity of key lipid metabolic enzymes, such as, SREBP-1c, C/EBPα, and FAS. Consequently, BDMC suppressed fat accumulation in the liver and could be developed as a potential therapeutic treatment to reduce formation of a fatty liver.
